# Large Human Outbreak of West Nile Virus Infection in North-Eastern Italy in 2012

**DOI:** 10.3390/v5112825

**Published:** 2013-11-22

**Authors:** Luisa Barzon, Monia Pacenti, Elisa Franchin, Silvana Pagni, Enrico Lavezzo, Laura Squarzon, Thomas Martello, Francesca Russo, Loredana Nicoletti, Giovanni Rezza, Concetta Castilletti, Maria Rosaria Capobianchi, Pasquale Salcuni, Margherita Cattai, Riccardo Cusinato, Giorgio Palù

**Affiliations:** 1 Department of Molecular Medicine, University of Padova, Padova I-35121, Italy; E-Mails: elisa.franchin@unipd.it (E.F.); silvana.pagni@unipd.it (S.P.); enrico.lavezzo@unipd.it (E.L.); laura.squarzon@unipd.it (L.S.); thomas.martello@unipd.it (T.M.); 2 Regional Reference Laboratory for Infectious Diseases, Microbiology and Virology Unit, Padova University Hospital, Padova I-35128, Italy; E-Mails: monia.pacenti@sanita.padova.it (M.P.); margherita.cattai@sanita.padova.it (M.C.); riccardo.cusinato@sanita.padova.it (R.C.); 3 Department of Public Health and Screening, Veneto Region, Venice I-30123, Italy; E-Mail: Francesca.Russo@regione.veneto.it; 4 Department of Infectious, Parasitic and Immune-mediated Diseases, National Institute of Health (Istituto Superiore di Sanità, ISS), Rome I-00161, Italy; E-Mails: loredana.nicoletti@iss.it (L.N.); giovanni.rezza@iss.it (G.R.); 5 National Institute for Infectious Diseases (INMI) “L. Spallanzani”, Rome I-00149, Italy; E-Mails: concetta.castilletti@inmi.it (C.C.); maria.capobianchi@inmi.it (M.R.C.); 6 Department of Prevention and Communication, Ministry of Health, Rome I-00144, Italy; E-Mail: p.salcuni@sanita.it

**Keywords:** West Nile virus, surveillance, West Nile neuroinvasive disease, West Nile fever, blood donor screening, molecular testing

## Abstract

Human cases of West Nile virus (WNV) disease have been reported in Italy since 2008. So far, most cases have been identified in north-eastern Italy, where, in 2012, the largest outbreak of WNV infection ever recorded in Italy occurred. Most cases of the 2012 outbreak were identified in the Veneto region, where a special surveillance plan for West Nile fever was in place. In this outbreak, 25 cases of West Nile neuroinvasive disease and 17 cases of fever were confirmed. In addition, 14 WNV RNA-positive blood donors were identified by screening of blood and organ donations and two cases of asymptomatic infection were diagnosed by active surveillance of subjects at risk of WNV exposure. Two cases of death due to WNND were reported. Molecular testing demonstrated the presence of WNV lineage 1 in all WNV RNA-positive patients and, in 15 cases, infection by the novel Livenza strain was ascertained. Surveillance in other Italian regions notified one case of neuroinvasive disease in the south of Italy and two cases in Sardinia. Integrated surveillance for WNV infection remains a public health priority in Italy and vector control activities have been strengthened in areas of WNV circulation.

## 1. Introduction

West Nile virus (WNV), a mosquito-borne flavivirus of the Japanese encephalitis sero-complex, is an emerging pathogen that is causing disease in humans in Europe and worldwide [1]. The virus is transmitted to a variety of vertebrate species through the bite of infected mosquitoes, mainly of the *Culex* genus. Birds play a key role in viral amplification, while mammals are generally dead-end hosts because of the low level WNV viraemia that can not support infection of mosquito vectors [2]. Most WNV infections in humans are asymptomatic or associated with mild symptoms; in approximately 20% of cases, WNV infection is associated with a febrile illness (defined as West Nile fever, WNF), characterized by fever, headache, fatigue, malaise, muscle pain, and, less frequently, gastrointestinal symptoms and skin rash; less than 1% of infected persons show neurological symptoms (defined as West Nile neuroinvasive disease, WNND) that may present as meningitis, encephalitis, or poliomyelitis-like acute flaccid paralysis [3,4]. Advanced age, immunosuppression and co-morbidities, like cancer, hypertension and diabetes, are recognized risk factors for severe disease [5].

The virus was isolated for the first time in Uganda in 1937 [6] and, initially, it was responsible for epidemic outbreaks in Africa and in the Middle East and for only sporadic cases of infection in Europe [7]. The fist large human outbreak of WNV infection recorded Europe occurred in Romania in 1996, with a high number of WNND cases and a high fatality rate [8], followed by other outbreaks and sporadic cases reported in Czech Republic, Southern Russia, and Hungary [9,10]. In the recent years, the virus has re-emerged in Europe and human cases of WNND have been notified in almost all Eastern, Central, and Southern European Countries [11,12], with most cases reported in Italy, since 2008 [13,14], and in Greece, since 2010 [15,16]. The virus has also spread to North America, where it was first introduced in 1999 [17] followed by a rapid propagation to all the US States and to other North American countries, where it has caused thousands of human infections each year [18]. Only WNV lineage 1 genotypes, all derived from the single introduction that occurred 1999, circulate in North America, while different WNV strains, belonging to both lineage 1 and lineage 2 and probably deriving from multiple independent introductions from Africa, are circulating and causing disease in Eurasia [19,20,21,22].

In Italy, circulation of different WNV genotypes of both lineage 1 and lineage 2 has been documented, even in the same area [23,24,25], and outbreaks in humans have been reported since 2008, when the first human cases of WNND were identified in areas surrounding the Po river in Northern Italy [26,27]. In the following years, cases of WNND were diagnosed in a larger area near the Po river [28,29,30], while sero-prevalence studies in blood and organ donors [31,32] and veterinary and entomological investigation indicated viral activity also in other regions [33]. This led the Italian Ministry of Health to publish in 2010 a national plan for WNND human surveillance that integrated veterinary and vector surveillance [13]. In addition, based on the evidence of widespread WNV circulation in its territory, Veneto region implemented a special surveillance program for WNF [34], besides surveillance for WNND according to the national plan. While in 2008–2010 human cases of infection were detected only in northeastern Italy and were caused by WNV lineage 1 genotypes [28,29,30,35], in 2011, in addition to northeastern Italy [23], cases were notified for the first time also in Marche region (Central Italy) and in Sardinia island and included infections with WNV lineage 2 [25,36].

These epidemiological data show that Italy is at risk for the occurrence of WNV outbreaks and indicate the importance of having in place surveillance programs for WNV infection in areas at risk. Indeed, the largest outbreak ever reported in Italy occurred in 2012 in north-eastern Italy and was rapidly recognized by the surveillance activity [37]. This outbreak as well as other cases of human infection were identified in Italy in 2012 by the national surveillance plan, and are described in this report.

## 2. Results and Discussion

### 2.1. Human Outbreak of WNV Infection in North-Eastern Italy, 2012

**Figure 1:**
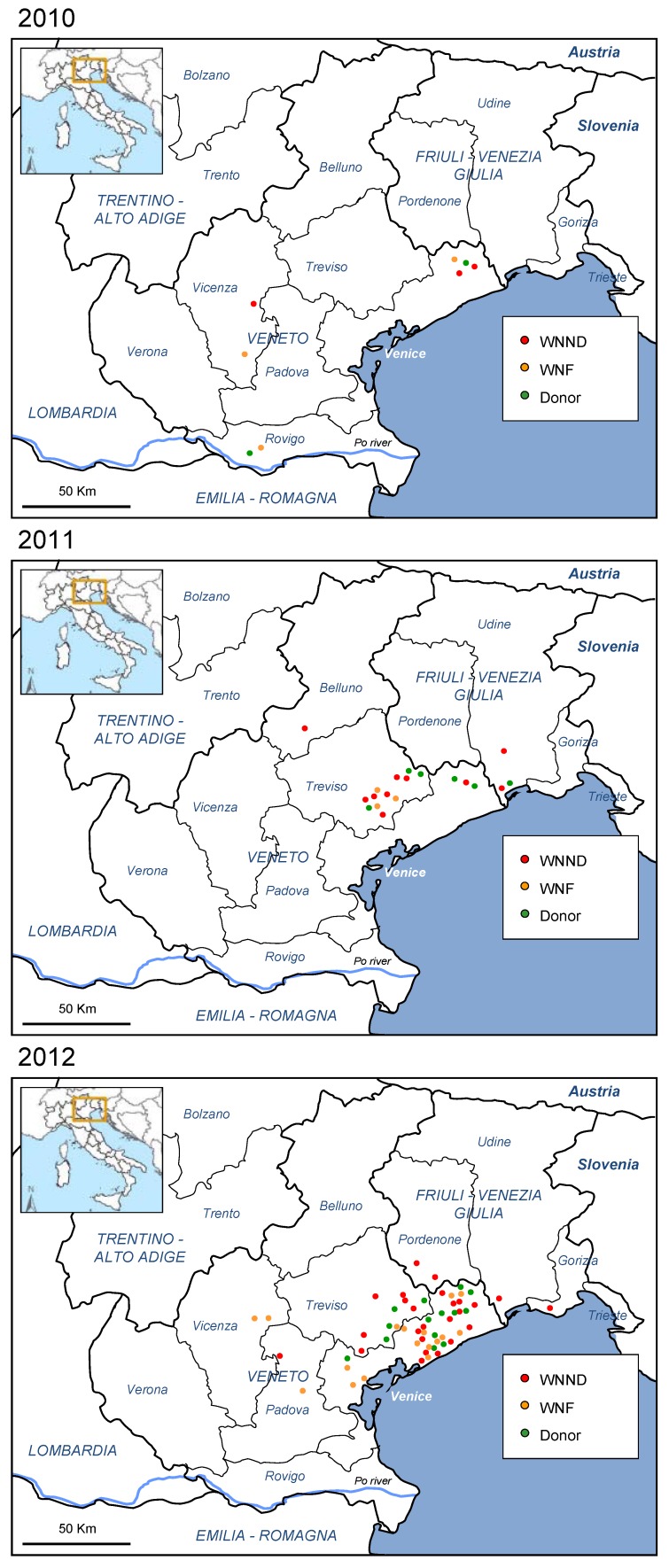
Map of human West Nile virus (WNV) cases of WNV infection confirmed in North-eastern Italy, 2010–2012. WNND: cases of West Nile neuroinvasive disease; WNF: cases of West Nile fever; Donor: blood, tissue, and organ donors with confirmed WNV infection.

**Figure 2:**
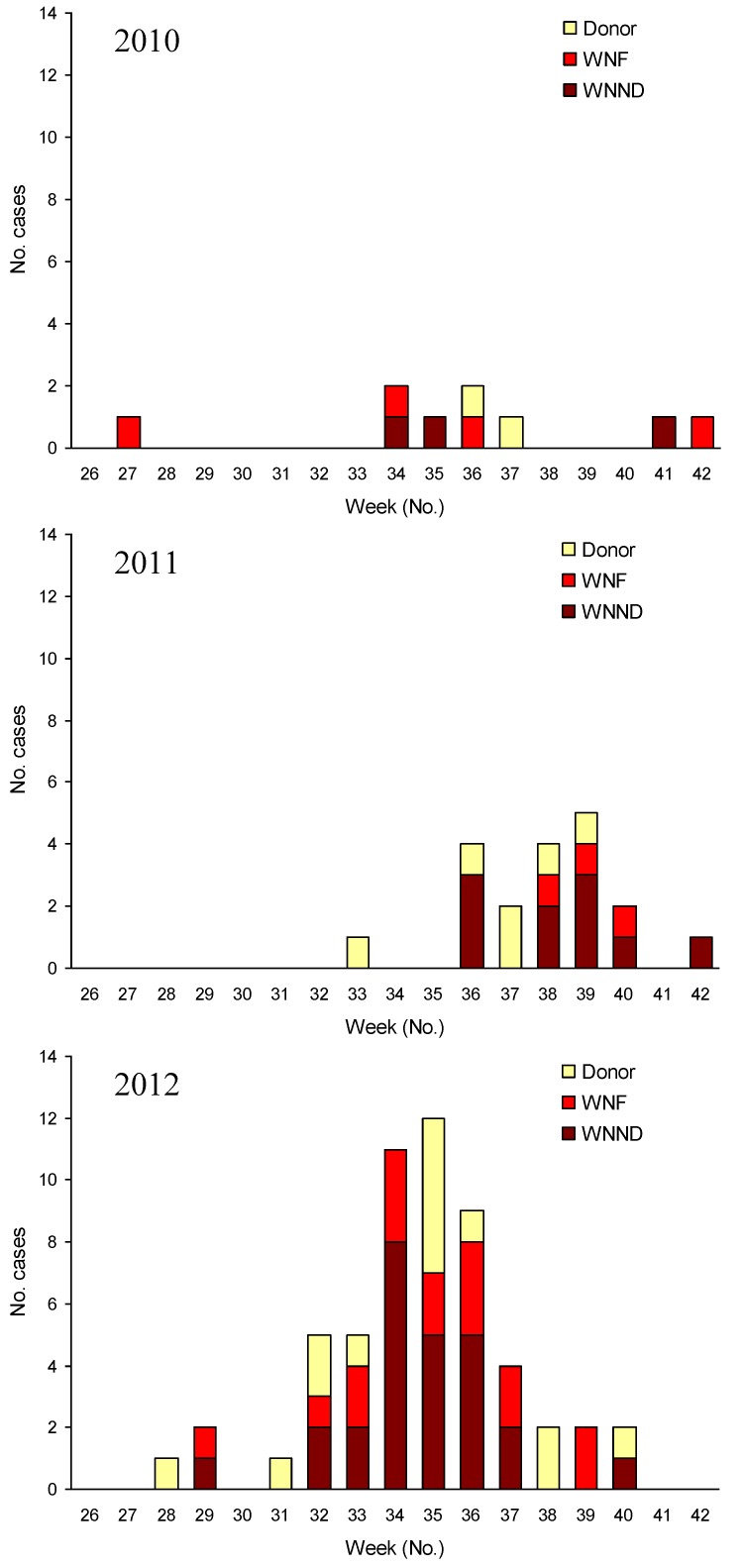
Cases of West Nile infection by week of symptom onset confirmed in North-eastern Italy, 2010–2012. WNND: cases of West Nile neuroinvasive disease; WNF: cases of West Nile fever; Donor: blood, tissue, and organ donors with confirmed WNV infection.

In 2012, most human cases of WNV infection occurred in north-eastern Italy, in areas that had been affected also in 2010 and 2011 (Figure 1) [13,23,35]. However, in 2012, a markedly higher incidence of WNV infection was observed than in previous years, with 25 cases of WNND, 17 cases of WNF, 14 WNV RNA-positive blood donors, and further two cases of asymptomatic WNV infection identified by active surveillance of subjects at risk of WNV exposure. In Venice province, where the highest number of human cases of WNV infection was diagnosed, the incidence of WNND and WNF was 1.65/100,000 and 1.41/100,000, respectively (annual incidence rates calculated using the 2010 population of Venice province available from the National Bureau of Statistics [38]), while the rate of WNV infection in screened blood donors was 52.14/100,000. The distribution over time of human cases of WNV infection by week of symptom onset is reported in Figure 2, which shows an earlier presentation and a higher number of WNV cases in 2012 than in previous years.

The outbreak occurred after an exceptionally warm spring and during a very hot summer season, in places characterized by a very high mosquito density. In 2012, such warm climate conditions were also observed in other European and Mediterranean Countries and in the United States, that reported earlier occurrence of an increased number of human cases of WNV infection to the European Centre for Disease Prevention and Control (a total of 907 cases in 2012, including 447 cases in the Russian Federation, 161 in Greece, 83 in Israel, 69 in Serbia, 63 in Tunisia, and smaller number of cases in other countries) [39] and to the Centers for Disease Control and Prevention (a total of 5,387 cases in 2012, most in whom reported in Texas) [40].

The median age of patients with WNND was 69 years (range, 10–90 years), including three patients with encephalitis younger than 30 years, while the median age of patients with WNF was 63 years (range, 45–80 years), and 57 years (range, 36–64 years) in WNV NAAT-positive blood donors.

Patients with WNND had meningitis in 8 cases, encephalitis in 12 cases, and acute flaccid paralysis in 5. As detailed in Table 1, symptoms like fever, headache, and fatigue were common both in patients with WNND and in those with WNF, while myalgia, and arthralgia were more frequent in patients with WNF than in those with WNND; skin rash and gastrointestinal symptoms were less frequently observed. Symptoms were reported also in 10 of the 14 blood donors with WNV infection, most commonly headache, fatigue, myalgia, and arthralgia that required hospitalization in one case. Two cases of death due to WNND were reported.

The results of laboratory tests at the time of diagnosis are reported in Table 2. The implementation of WNV RNA testing in urine for the routine diagnosis of WNV infection was a useful method that allowed the rapid confirmation of WNV cases and the characterization of WNV nucleic acid sequences in a relatively high number of patients. In fact, assessment of the presence of WNV RNA in urine samples by real-time RT-PCR was more sensitive than testing WNV RNA in plasma or CSF, with a higher rate of positive results. WNV RNA was present in urine at higher load and for longer periods than in plasma. In fact, in some of the patients of this study, the presence of WNV RNA in urine was found at a distance of one month after the onset of symptoms, when specific IgM and IgG were already present in serum and the patient was no longer viraemic, as reported [14,41,42]. In patients with WNND, the search for WNV RNA in CSF was not proven to be a sensitive test, while WNV IgM testing in CSF was very sensitive, but required confirmation by neutralization test.

WNV subtyping by PCR and sequencing of a NS5 region was successful in 11 patients with WNND and WNF and in four blood donors and demonstrated infection by the WNV lineage 1 Livenza strain in all cases. In another eight patients with a positive molecular test, infection by WNV lineage 1 was ascertained by using lineage-specific real-time RT-PCR assays.

**Table 1:** Frequency of symptoms and signs in patients with WNV infection, Northeastern Italy, 2012.

Symptoms and signs	WNND	WNF	Blood donor
	(*n* = 25)	(*n* = 17)	(*n* = 14)
Fever	80%	100%	14%
Headache	68%	71%	21%
Fatigue	60%	59%	64%
Rash	0%	18%	14%
Artralgia	8%	47%	21%
Myalgia	20%	4%	29%
Lymphoadenopathy	0%	6%	0%
Vomiting/diarrhea	0%	12%	21%
Neurological manifestations	68%	0%	7%
Respiratory failure	24%	0%	0%
Encephalitis	48%	0%	0%
Meningitis	32%	0%	0%
Acute flaccid paresis/paralysis	20%	0%	0%
Asymptomatic	0%	0%	29%

WNND: patients with WNV neuroinvasive disease; WNF: patients with WNV fever; blood donor: patients in whom WNV infection was identified by WNV NAAT screening of blood donation.

**Table 2:** Summary of the results of virological tests performed in confirmed cases of WNV infection, Northeastern Italy, 2012. (minus sign or not? OK, it is minus sign)

	WNND (*n* = 25)	WNF (*n* = 17)	Blood donors (*n* = 14)
Parameter	No. Positive/No. Tested (%)	No. Positive/No. Tested (%)	No. Positive/No. Tested (%)
WNV RNA in plasma	7/22 (31.8)	3/17 (17.6)	8/14 (57.1)
WNV RNA in urine	7/16 (43.8)	7/17 (41.1)	2/14 (14.3)
WNV RNA in CSF	1/19 (5.3)	0/2 (0)	0/0
Serum IgM-/IgG-	0/25 (0)	0/17 (0)	2/14 (14.3)
Serum IgM+/IgG-	6/25 (24.0)	3/17 (17.6)	6/14 (42.9)
Serum IgM+/IgG+	19/25 (76.0)	14/17 (82.3)	6/14 (42.9)
CSF IgM+/IgG-	6/19 (31.6)	0/0	0/0
CSF IgM+/IgG+	13/19 (68.4)	0/0	0/0

The reported laboratory test results were performed on the first blood, CSF, and urine samples collected from patients with WNND or WNF and collected from blood donors at the first follow-up visit after blood donation. WNND: patients with WNV neuroinvasive disease; WNF: patients with WNV fever; blood donor: patients in whom WNV infection was identified by WNV NAAT screening of blood donation; CSF: cerebrospinal fluid.

**Figure 3:**
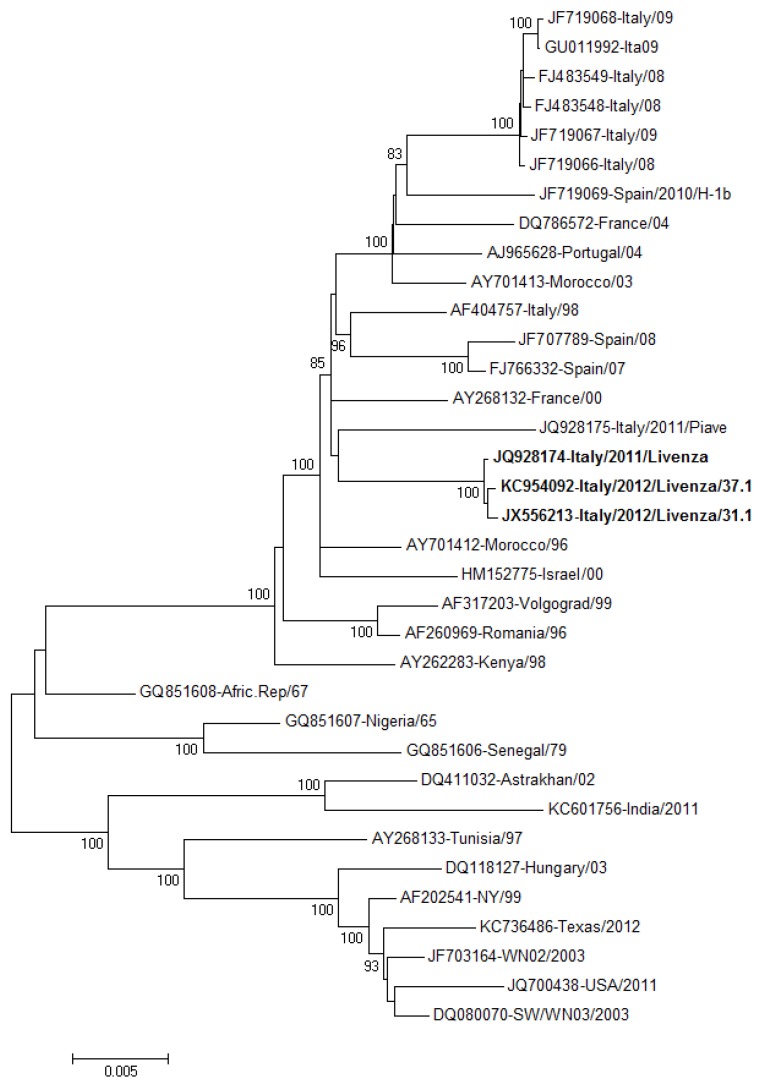
Molecular Phylogenetic analysis of WNV lineage 1 whole genome sequences by Maximum Likelihood method. The evolutionary history was inferred by using the Maximum Likelihood method based on the Kimura 2-parameter model [43]. The percentage of trees in which the associated strains clustered together is shown next to the branches (only for values ≥ 80). The tree is drawn to scale, with branch lengths measured in the number of substitutions per site. The analysis involved 35 nucleotide sequences. All positions containing gaps and missing data were eliminated. There were a total of 10,385 positions in the final dataset. Evolutionary analyses were conducted in MEGA5 [44].

The virus was isolated in Vero cell cultures from serum of a blood donor and from urine of a patient with WNND. Whole genome sequencing of these two isolates (GenBank accession numbers JX556213.1 and KC954092, respectively) demonstrated 99.9% nucleotide sequence identity with each other and with the WNV Livenza strain that was fully sequenced in the same area in 2011 (GenBank accession number JQ928174.1) [23]. Comparison among viral polyprotein amino acid sequences demonstrated only neutral changes (e.g., Val118Ile and Val113Ile in the capsid and Val45Ile in the NS5 protein) that probably did not affect viral fitness or pathogenicity. A phylogenetic tree including the three fully sequenced WNV Livenza genomes and their relationship with other WNV strains of the Western Mediterranean subtype is represented in Figure 3. As shown in the figure, the WNV Livenza strain is included in the group of Mediterranean WNV Lineage 1 genotypes, but has no close sequence similarity with other fully sequenced genomes of this group.

In the same areas in northeastern Italy where human cases were recorded, the WNV lineage 1 Livenza strain was detected in mosquitoes pools collected for entomological surveillance, while a PCR positivity for WNV lineage 2 related to the Greek strain (GenBank accession no. JX878386.1 and JX878387.1) was demonstrated in a mosquito pool collected in a site near the Po river, in a province where no human cases of WNV infection were reported in 2012 [45].

The other WNV lineage 1 genotype that was detected in the same areas in 2011 [23] and the WNV lineage 1 strain that was responsible for the large outbreak that occurred in Veneto and Emilia-Romagna regions in 2008–2009 [29,46] were not detected in 2012 by human, veterinary, or entomological surveillance. It is conceivable that the genetic background of the WNV Livenza strain, together with suitable local ecological conditions, have been relevant for its overwintering and spread in a relatively large area in north-eastern Italy in 2012.

### 2.2. Human Cases of WNV Infection in Other Italian Regions, 2012

In addition to the outbreak in north-eastern Italy, 3 cases of WNND were notified in other Italian regions, including 1 case from Basilicata region (Matera Province) in the South of Italy who had symptom onset at the end of September, and 2 cases from Sardinia (Oristano Province). These cases had WNV specific IgM in serum and CSF, confirmed by neutralization test, while no WNV-RNA was detected in serum, CSF, or urine. Of note, the patient from Basilicata region had undergone kidney transplantation ten years before.

In these regions, veterinary and entomological surveillance documented the circulation of WNV, with evidence of the widespread presence of WNV lineage 2 in Sardinia island [45,47].

## 3. Experimental Section

### 3.1. National Plan for WNND Surveillance in Humans, Italy, 2012

Surveillance for autochthonous human cases of WNND was carried out in affected areas and in surrounding surveillance areas between 15 June and 30 November 2012, according to the Italian National Plan for WNND Human Surveillance [48]. Briefly, the national plan for human surveillance defined the “area with virus circulation”, where laboratory-confirmed WNV infections in horses or humans had been notified in previous years or during the surveillance period, and the “surveillance area external to the area with virus circulation” that extended for a 20 km radius around the cases occurring in the outermost parts of the area with virus circulation.

Activities included passive surveillance of human cases of WNND, which was carried on in areas with virus circulation and in surveillance areas, and active surveillance of WNV infection, which was carried on in areas with virus circulation in stable workers employed in farms where equine cases of WNV infection had been identified and in household contacts of patients with WNND, as described in the following sections.

### 3.2. Special Surveillance Plan for West Nile Fever in Humans, Veneto Region, Italy, 2012

Based on the evidence of widespread WNV circulation in the regional territory, Veneto region (in northeastern Italy) also implemented a surveillance plan for WNF since 2010 [23,34]. According to this surveillance plan, all patients with unexplained fever over 38 °C and without leukocytosis were considered possible cases of WNF and were investigated for WNV infection with laboratory tests.

### 3.3. Case Definition of WNND and WNF

Case definition of WNND and WNV was according to ECDC criteria [49], as previously reported [13,35], with the introduction of WNV isolation or nucleic acid detection in urine among confirmation criteria [48].

### 3.4. Case Laboratory Investigations

Possible cases of WNND and WNF were investigated with the following laboratory tests.

Detection of WNV RNA in plasma, urine, and CSF samples by using two different real-time RT-PCR methods, targeting WNV lineage 1 [50] and both WNV lineage 1 and lineage 2 [51], and by a pan-flavivirus nested RT-PCR assay followed by cycle sequencing [52].

Detection of IgM and IgG antibodies against WNV in serum and CSF samples was done by ELISA (WNV IgM capture DxSelect ELISA and IgG DxSelect ELISA kits, Focus Diagnostics, Cypress, CA, USA). To confirm the specificity of antibody response, ELISA-positive samples were further tested by PRNT90, with cutoff 1:10 for positive results, as described [35].

### 3.5. Active Surveillance of Stable Workers and Household Contacts

Active surveillance of WNV infection was done for workers employed in farms and for subjects residing in farms where equine cases of WNV infection had been identified. Members of households with confirmed cases of WNND or WNF and close contacts of identified human or equine cases of WNV disease were also surveyed. Laboratory workup included testing for WNV RNA in plasma samples by real-time RT-PCR and IgM and IgG antibodies against WNV in serum samples by ELISA and confirmation by PRNT, using above described methods.

### 3.6. Screening of Blood and Organ Donations

In 2012, according to the National Blood Directive and the National Transplant Coordination, NAAT screening was performed in the period between 15 July to 30 November for all hematopoietic stem cells and blood donations in provinces where human cases of WNND had been identified in the previous year and in provinces where new human cases of infection were identified in 2012, while screening of tissue and organ donations was performed in regions affected by human cases in 2011 or with new cases identified in 2012. Deferral of donations was applied to potential blood donors for a period of 28 days after leaving an affected area with ongoing transmission of WNV to humans. WNV NAAT screening of blood, tissue, and organ donors was performed by using the Cobas TaqScreen West Nile Virus test on Cobas S201 system (Roche Molecular Diagnostics, Pleasanton, CA, USA) or the Procleix WNV Assay on Procleix Tigris System (Novartis Diagnostics, Emeryville, CA, USA). WNV NAAT-positive blood donors were referred to Reference Laboratories for confirmation and followed up at 7, 21, 40 days and at 6 months from donation for clinical and laboratory evaluation. 

## 4. Conclusions

This study, which reports human cases of WNV infection notified in Italy in 2012, describes an outbreak with 56 confirmed cases of WNV infection, including 25 cases of WNND and 17 cases of WNF, representing the largest WNV outbreak in humans ever recorded in Italy. The outbreak occurred after an exceptionally warm spring and during a very hot summer season in a relatively small area in northeastern Italy that was affected by human cases of WNV infection also in 2010 and 2011. Whole genome sequencing of two viral isolates demonstrated very high sequence similarity with the WNV Livenza genome that was fully sequenced in 2011 [23].

A special surveillance plan for WNF and other vector-borne diseases was in place in Veneto region, which was the most affected region. This plan certainly contributed to the efficient and rapid identification of cases of WNV infection.

To face the risk of a new WNV epidemic in 2013, Regional Health Departments, in collaboration with Local Public Health Departments, have planned strengthened public health interventions with enhanced active surveillance and vector mosquito control plans in affected municipalities and with more diffused and detailed information to the general population on how WNV infection is acquired and on the use personal protective practices. 
